# Concurrent Detection of Swine-Origin Influenza A(H1N1) Virus in Pigs and Farmer, Switzerland

**DOI:** 10.3201/eid3206.251487

**Published:** 2026-06

**Authors:** Jonas Steiner, Mike Mwanga, Larise Oberholster, Matthias Licheri, Manon F. Licheri, Heiko Nathues, Ronald Dijkman, Jenna N. Kelly

**Affiliations:** University of Bern Multidisciplinary Center for Infectious Diseases, Bern, Switzerland (J. Steiner, M. Mwanga, L. Oberholster, M. Licheri, M.F. Licheri, H. Nathues, R. Dijkman, J.N. Kelly); University of Bern Vetsuisse Faculty, Bern (J. Steiner, M. Mwanga, H. Nathues, J.N. Kelly); University of Bern Faculty of Medicine, Bern (M. Mwanga, L. Oberholster, M. Licheri, M.F. Licheri, R. Dijkman); Institute of Virology and Immunology, Bern and Mittelhäusern, Switzerland (M. Mwanga, J.N. Kelly)

**Keywords:** influenza A virus, swine, swine flu, influenza, viruses, respiratory infections, surveillance, molecular epidemiology, genomics, zoonoses, whole-genome sequencing, Switzerland

## Abstract

We report zoonotic transmission of Eurasian avian-like swine influenza A(H1N1) virus from pigs to a farmer. The pigs and farmer experienced influenza-like illness. Whole-genome sequencing revealed >99.9% viral sequence identity between hosts. Our findings highlight the risk posed by enzootic swine influenza A virus and the need for genomic and epidemiologic surveillance.

Since the 2009 swine influenza pandemic, sporadic human infections with swine influenza A viruses (swIAVs) continue to occur, including rare instances of onward human-to-human transmission, highlighting the ongoing pandemic risk (*1*–*4*). Pigs are key reservoirs and mixing vessels for influenza A virus (IAV) evolution; transmission between humans and pigs is frequent and bidirectional (*5*,*6*). Active epidemiologic and genomic surveillance at the swine–human interface is therefore essential for early detection and risk assessment of emerging strains.

In Switzerland, pig production is less intensive; herds remain relatively isolated from neighboring countries because of strict regulations and minimal import of live pigs (*7*,*8*). Infrequent use of swIAV vaccines allows for natural viral evolution without vaccine-driven selective pressures (*9*); country-specific transmission chains may exist within pig herds in Switzerland. The ongoing national surveillance program for swIAV relies primarily on partial hemagglutinin (HA) and neuraminidase (NA) gene sequences, limiting its ability to identify emerging swIAV lineages in pig herds (*10*).

To investigate the epidemiology and genetic diversity of swIAV in pig herds, we established a whole-genome sequencing (WGS)–based swIAV surveillance program. We obtained samples from symptomatic and randomly selected pig herds; pig caretakers with respiratory illness voluntarily provided self-collected nasal swab specimens. Here, we report a zoonotic transmission event involving Eurasian avian-like (EA) swine influenza A(H1N1) virus detected concurrently in a farmer and his pig herd through WGS surveillance.

## The Study

On November 27, 2023, a respiratory disease outbreak was reported in a herd of »180 fattening pigs, 4 weeks after the introduction of 90 growing pigs from another herd from within Switzerland. Upon clinical examination, »80% of pigs showed apathy and fever (<40.3°C) and sporadic respiratory signs. The farmer, who was 40–50 years of age with no reported underlying conditions, experienced a mild influenza-like illness 2 weeks earlier and reported similar symptoms in 2 household members. The farmer was not vaccinated against seasonal influenza and reported no contact with other pig herds.

**Figure 1:**
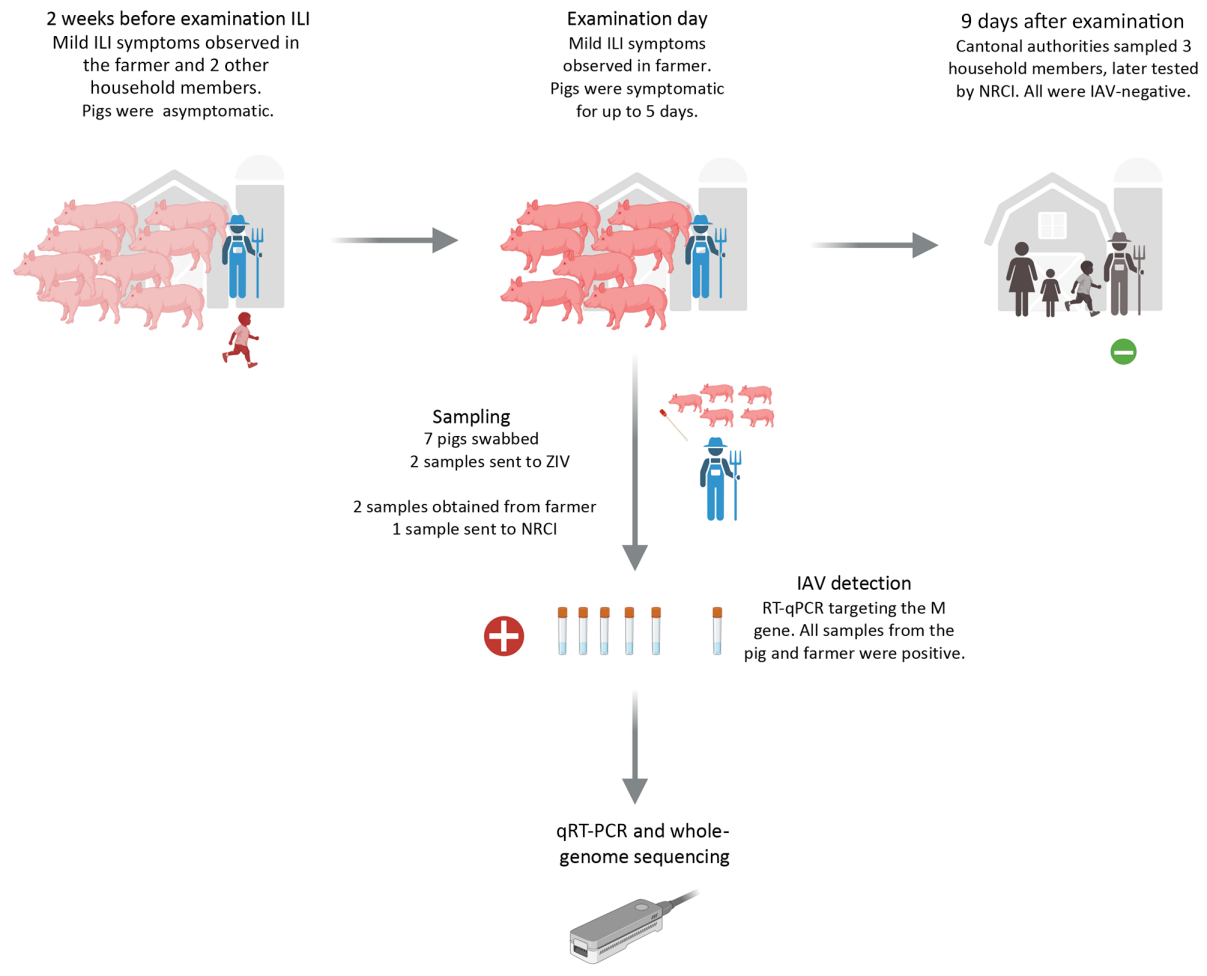
Timeline of events from study of swine-origin influenza A(H1N1) virus in pigs and farmer, Switzerland, November 2023. Image depicts the chronology of symptoms in pigs and humans, sampling dates, and household investigations, along with corresponding qRT-PCR results. Figure created in BioRender (Mwanga M, 2025, https://BioRender.com/te6k5rt). IAV, influenza A virus; ILI, influenza-like illness; NRCI, National Reference Centre for Influenza, Switzerland; M, matrix; qRT-PCR, quantitative reverse transcription PCR; ZIV, Institute of Virology, Vetsuisse Faculty, University of Zurich; –, negative; +, positive.

We collected nasal swab samples from pigs and the farmer through our WGS-based surveillance program and the national swIAV surveillance system. Within the WGS surveillance framework, we screened 5 pig samples (A0001–A0005) and 1 farmer sample (H0001) for IAV using a pan-IAV matrix gene–specific quantitative reverse transcription PCR assay on extracted viral RNA (Appendix Table 1) (*11*). All samples tested positive; viral loads were higher in pigs (crossing point [Cp] value 24–26) than in the farmer sample (Cp 32) (Table 1). After confirming variant H1N1 (H1N1v) infection in the farmer, we notified veterinary and public health authorities and reported the case through the World Health Organization National Focal Point in accordance. Nine days after case confirmation, nasal swabs from all 3 household contacts tested negative for IAV at the Swiss National Reference Centre for Influenza (NRCI) (Figure 1) (A.R. Gonçalves Cabecinhas, pers. comm., email, 2024 Dec 2).

**Table 1:** Sample and sequencing data collected in study of influenza A(H1N1) virus in pigs and farmer, Switzerland, November 2023*

Sample	Host	Life stage*	Crossing point value	Total no. reads	No. (%) viral reads
A0001	Pig	Fattening pig	24.60	454,678	448,020 (98.5)
A0002	Pig	Fattening pig	25.76	276,888	271,662 (98.1)
A0003	Pig	Fattening pig	23.79	571,358	563,098 (98.5)
A0004	Pig	Fattening pig	25.10	358,462	353,591 (98.6)
A0005	Pig	Fattening pig	26.07	1,173,972	1,161,694 (98.9)
H0001	Human	Adult	32.47	10,779	8,174 (74.6)

*Life stage for pigs is »22 wk; for human adult, 40–50 y.

**Figure 2:**
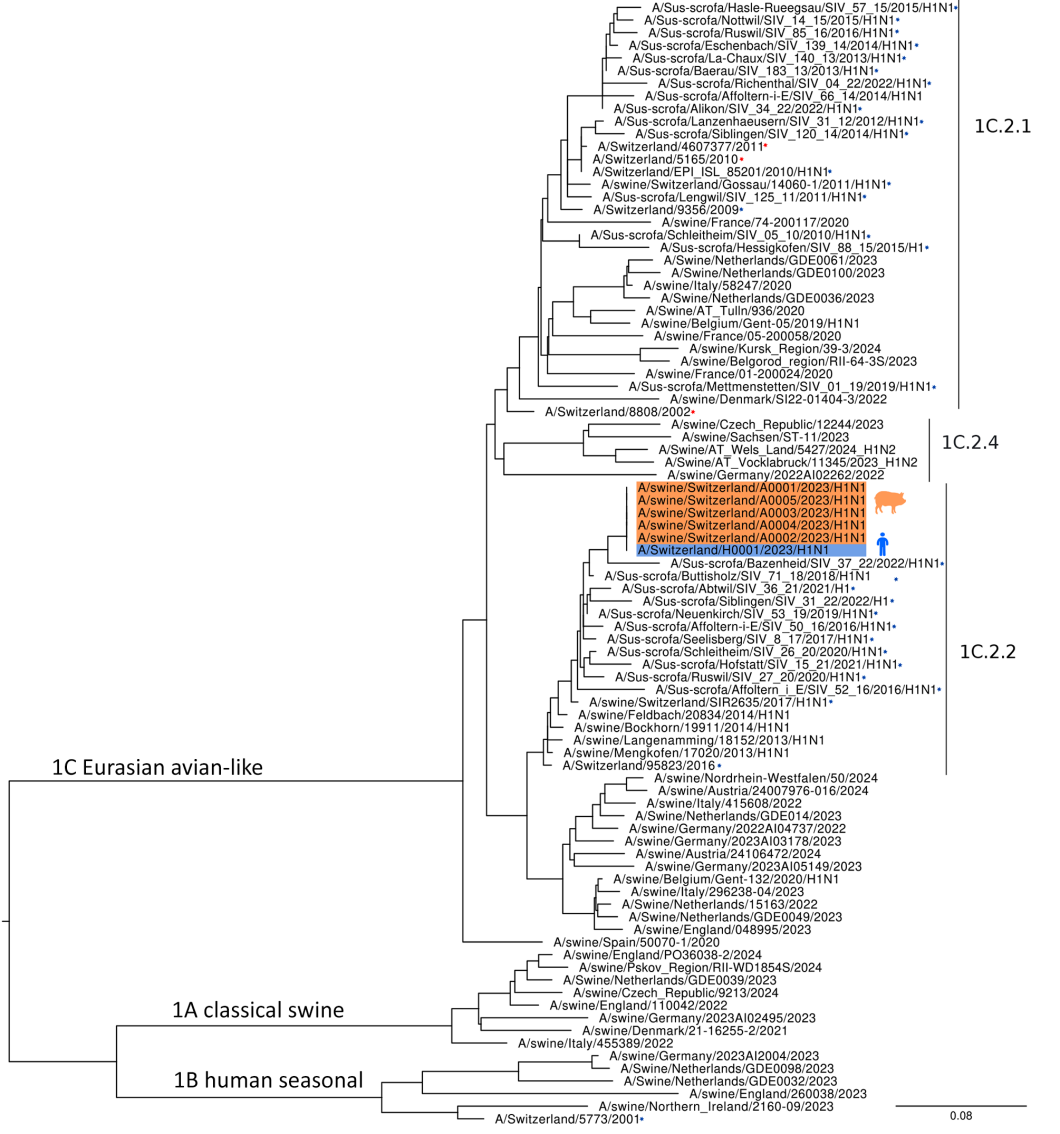
Maximum-likelihood phylogenetic tree from study of swine-origin influenza A(H1N1) virus in pigs and farmer, Switzerland, November 2023. We mapped hemagglutinin (HA) gene codon sequences, showing lineage classification of the study swine H1N1 virus based on lineage reference sequences and HA-H1 sequences from viruses identified in Switzerland and other Europe countries (2020–2023). The tree shows classification based on the 3 major swine H1N1 lineages: 1A, 1B, and 1C. Orange shading indicates swine H1N1 HA sequences from pig samples; blue indicates sequence from the farmer. Other swine H1N1 HA sequences from Switzerland are marked with asterisks: blue for those identified in pig samples and red for those identified in human samples. Study sequences cluster within the Eurasian avian-like lineage clade 1C.2.2. Pig sequences from previous years are grouped in clades 1C.2.1. and 1C.2.2. Scale bar indicates substitutions per site.

We performed WGS as described previously (*12*), achieving high read depth (>1,000´) overall; 1 segment showed lower coverage (>100´), sufficient for complete genome assembly (Table 1; Appendix Table 1, Figure 1). We subtyped all viruses from human and pig samples as swine H1N1 (swH1N1); nucleotide identity was >99.9% across all genomic segments. Phylogenetic analysis with publicly available swH1N1 sequences from Switzerland and Europe showed that all study viruses were assigned to the EA swH1N1 lineage, clade 1C.2.2, forming a monophyletic cluster for all genomic segments (Figure 2; Appendix Figure 2). Similar to other EA swH1N1 viruses, the Switzerland sequences harbored several amino acid substitutions associated with host specificity and antiviral resistance (Appendix Table 2).

We assessed low-frequency diversity by variant calling across all genomic segments. We detected 26 minor single-nucleotide variants (SNVs) at 6%–50% frequency, resulting in 17 nonsynonymous mutations. We detected 4 human-specific SNVs, including C1771T (H0001), which resulted in a premature stop codon in the polymerase acidic (PA) gene (Q591*). We identified 4 SNVs shared between human and pig samples, including G515A, which resulted in the HA-G172E mutation associated with antigenic drift and potential immune escape (Appendix Table 3, Figure 3) (*13*). We detected 1 SNV (PB2–1447), leading to a T483A mutation, in all samples. The presence of shared SNVs supports epidemiologic linkage between the farmer and pigs.

Finally, we compared HA antigenic residues of study viruses to the 2023–24 human seasonal vaccine strain and a contemporaneous (2023) human H1N1 sequence (Table 2). We identified multiple substitutions in the swH1N1 sequences across defined antigenic sites Sa/Sb, Ca1, Ca2, and Cb; the contemporaneous human seasonal virus differed from the vaccine strain by 2 antigenic residues. Overall, swH1N1 sequences from pigs and the farmer showed greater divergence at HA antigenic sites relative to the vaccine strain than the contemporaneous human seasonal virus, consistent with their evolutionary divergence from human H1N1 viruses.

**Table 2:** Comparison of amino acid sequences in the hemagglutinin antigenic region of swine H1N1, human seasonal H1N1, and seasonal vaccine strains in study of influenza A(H1N1) virus in pigs and farmer, Switzerland, November 2023*

Isolate	HA-H1 residue positions												
Antigenic site Sa	124	125	153	154	155	156	157	159	160	161	162	163	164
Vaccine A/Wisconsin/67/2022	P	N	K	K	G	K	S	P	K	I	N	Q	T
Vaccine A/Victoria/4897/2022/H1N1	P	N	K	K	G	K	S	P	K	I	N	Q	T
Study A/Switzerland/H0001/2023/H1N1	P	N	K	K	G	N	A	P	K	I	R	K	S
Human seasonal H1N1	P	N	K	K	G	K	S	P	K	I	N	Q	T
Antigenic site Sb	184	185	186	187	188	189	190	191	192	193	194	195	
Vaccine A/Wisconsin/67/2022	T	I	T	D	Q	E	S	L	Y	Q	N	A	
Vaccine A/Victoria/4897/2022/H1N1	T	I	T	D	Q	E	S	L	Y	Q	N	A	
Study A/Switzerland/H0001/2023/H1N1	T	D	S	D	Q	Q	T	L	Y	Q	N	N	
Human seasonal H1N1	T	I	T	D	Q	E	S	L	Y	Q	N	A	
Antigenic site Ca1	166	167	168	169	170	203	204	205	235	236	237		
Vaccine A/Wisconsin/67/2022	I	N	D	K	G	T	S	R	E	P	G		
Vaccine A/Victoria/4897/2022/H1N1	I	N	D	K	G	T	S	R	E	P	G		
Study A/Switzerland/H0001/2023/H1N1	T	N	N	K	G	S	S	K	D	Q	G		
Human seasonal H1N1	I	N	D	Q	G	T	S	R	E	P	G		
Antigenic site Ca2	137	138	139	140	141	142	221	222					
Vaccine A/Wisconsin/67/2022	S	H	A	G	A	R	R	D					
Vaccine A/Victoria/4897/2022/H1N1	S	H	A	G	A	R	R	D					
Study A/Switzerland/H0001/2023/H1N1	S	H	S	G	T	K	R	E					
Human seasonal H1N1	P	H	A	G	A	K	R	D					
Antigenic site Cb	70	71	72	73	74	75							
Vaccine A/Wisconsin/67/2022	L	S	T	A	R	S							
Vaccine A/Victoria/4897/2022/H1N1	L	S	T	A	R	S							
Study A/Switzerland/H0001/2023/H1N1	L	L	T	A	D	S							
Human seasonal H1N1	L	S	T	A	R	S							

*Comparison of amino acid sequences in the antigenic region among the human 2023–24 seasonal vaccine strains, the swine H1N1 (swH1N1) virus identified in this study, and a representative human seasonal H1N1 virus. Numbering is based on the recommended HA-H1 numbering scheme using the HA Subtype Numbering Conversion tool (https://www.bv-brc.org/app/HASubtypeNumberingConversion). Orange shading denotes mutations distinguishing the vaccine strain from the study swine H1N1 virus. Gray shading indicates mutations distinguishing the vaccine strain from the representative human seasonal virus. A, alanine; D, aspartic acid; E, glutamic acid; G, glycine; H, histidine; HA, hemagglutinin; I, isoleucine; K, lysine; L, leucine; N, asparagine; P, proline; Q, glutamine; R, arginine; S, serine; T, threonine; Y, tyrosine.

## Conclusions

This study reports the concurrent detection of EA swH1N1 virus in a farmer and his pigs in Switzerland. Evidence for zoonotic transmission is >99.9% genomic identity between viruses from the farmer and pigs, shared SNVs between hosts, and epidemiologic data consistent with transmission at the swine–human interface. We identified several human-specific SNVs, but their functional significance remains unknown. We observed 1 SNV, PA C1771T (Q591*), at the consensus level in 1 pig sample; however, we cannot infer transmission direction or functional significance on the basis of the available data. The lower viral load we observed in the farmer sample may reflect previous immunity, timing of sampling, host-specific constraints, or the effects of the detected SNVs. We detected no onward transmission to household contacts; we could not exclude the possibility of secondary transmission because of the timing of sample collection. 

Genomic analysis revealed that the swH1N1 viruses identified in this study clustered with previously detected Switzerland sequences belonging to the EA lineage, clade 1C.2.2 (*10*). Despite limited availability of complete swH1N1 genome sequences from Switzerland, our findings are consistent with the presence of a relatively homogeneous swIAV population in pigs, likely shaped by the closed herd system and low annual importation of live pigs. Similarly, in Norway, the circulation of a single lineage under a closed system highlighted the effect of production structure on viral evolution (*14*). In contrast, greater swIAV genetic diversity has been reported in other countries in Europe (*15*). 

HA sequences of study swH1N1 viruses harbored multiple amino acid substitutions across defined antigenic regions, whereas contemporary human H1N1 strains differed from vaccine strains by only 1 or 2 residues. The detection of 1 SNV associated with HA antigenic drift highlighted hidden evolution; that finding suggests potentially limited cross-protection from human vaccines for exposed workers, underscoring the need for targeted vaccination strategies for high-risk occupational groups. Our investigation was restricted to 1 outbreak and a limited number of samples; we used no serologic data and only partial comparative sequences from Switzerland, which limited our assessment of evolutionary viral dynamics. Despite those constraints, the clinical, epidemiologic, and genomic evidence supports zoonotic transmission.

Concurrent detection of swIAV in pigs and humans is rare globally. The detection of EA swH1N1 in pigs and a farmer in our study demonstrates zoonotic spillover and highlights the value of One Health surveillance. The viruses were genetically homogenous, yet antigenically distinct from contemporary human vaccine strains, emphasizing the need for active WGS surveillance and genomic characterization at the swine–human interface, as well as continued monitoring and targeted preventive strategies for pig-exposed workers. 

Appendix Additional information about swine-origin influenza A(H1N1) virus in pigs and farmer, Switzerland, November 2023.
