# Supplementary material for: Concurrent Detection of Swine-Origin Influenza A(H1N1) Virus in Pigs and Farmer, Switzerland
Source: Emerg Infect Dis. 2026 Jun;32(6):975–80. doi: 10.3201/eid3206.251487 (PMC13245240; doi:10.3201/eid3206.251487)
Supplement: Appendix — Additional information about swine-origin influenza A(H1N1) virus in pigs and farmer, Switzerland, November 2023. [file 25-1487-Techapp-s1.pdf]

Article DOI: https://doi.org/10.3201/eid3206.251487

EID cannot ensure accessibility for supplementary materials supplied by authors. Readers who have difficulty accessing supplementary content should contact the authors for assistance.

Concurrent Detection of Swine-Origin Influenza A(H1N1) Virus in Pigs and Farmer, Switzerland

Appendix

Extended Methods

Study Description

To investigate the epidemiology and genetic diversity of swIAV in Swiss pig herds and identify strains with zoonotic potential, we established an active whole-genome sequencing (WGS) surveillance program. This program monitors an average of ten randomly selected healthy pig herds monthly and includes herds reporting respiratory disease outbreaks to private veterinarians or the Swiss Pig Health Service (SUISAG-SGD). For each reported herd, in addition to sampling for the active WGS surveillance program, ten additional samples were collected to exclude enzootic pneumonia, and two additional samples were collected for the passive national swIAV surveillance program (*1*).

During each inspection, a comprehensive herd examination and nasal sampling of pigs were performed by a licensed veterinarian. This included the recording of information related to husbandry practices and clinical presentation of the respiratory disease outbreak (if applicable). Corresponding nasal swabs were collected from pigs (FLOQSwabs ® Minitip; Copan, Brescia, Italy) by the licensed veterinarian, according to good veterinary practice standards. The sample size was calculated using WinEpi (http://www.winepi.net/uk/index.htm) to ensure detection of disease in at least one animal with 95% confidence at ≥50% within-herd prevalence. In cases of respiratory disease outbreaks, five pigs showing preferably acute clinical signs of fever and/or apathy were sampled. In addition to the pig sampling, caretakers with Influenza-like illness were invited to voluntarily provide a self-collected nasal swab specimen and to willingly disclose

other non-specific characteristics (e.g., smoker status, general health conditions, influenza vaccination status). All nasal swabs were transferred into a tube containing NucleoProtect® medium (Marchery-Nagel, Düren, Germany), transported at ambient temperature, and stored at ≥-20°C until further processing.

In the case described here, after the inspection, seven pigs were sampled by a licensed veterinarian – five for the active WGS surveillance program and two for the national passive surveillance program – and the farmer voluntarily submitted two self-collected nasal swabs. Of these, the five pig samples for the active WGS program and one of the farmer's samples were tested and sequenced at the Institute for Infectious Diseases (IFIK), University of Bern. An additional sample from the same farmer was analyzed at the Swiss National Reference Centre for Influenza (NRCI) as part of the national surveillance system, which also conducted follow-up human sampling and testing of the farmer and three household contacts (Figure 1).

This study is registered under the Swiss national animal experiment number 35801. The farmer provided written informed consent for the sampling of his pig herd. For data reporting, we consulted the CARE case report guidelines.

Detection of IAV by RT-qPCR

Viral RNA was extracted from nasal swab samples (five from pigs and one from the farmer) was performed as previously described (2). Detection of influenza A virus RNA was performed using the pan-IAV matrix segment-specific assay (3). Reactions were carried out in a final volume of 20 µl, consisting of 4.0 µl Reaction Mix, 0.1 µl Enzyme Solution from the LightCycler Multiplex RNA Virus Master (Roche, Basel, Switzerland), 0.8 µl of each primer, 0.2 µl probe, 2.0 µl of extracted RNA, and 12.1 µl of molecular grade H_2O. Reaction conditions are summarized in Appendix Table 1.

Library Preparation and Whole Genome Sequencing

Reverse transcription and PCR amplification were performed as previously described using the native barcoding approach (*3*). Each sample was processed in triplicate, and sequencing amplicons were prepared as previously described (*3*). Prior to library preparation, amplicons were size-selected to retain fragments >500 bp using AMPure XP beads (Beckman Coulter, Brea, CA, USA) at a 0.5× bead-to-sample ratio. Individual sequencing libraries were prepared using the ligation-based native barcoding kit (SQK-NBD114.24; Oxford Nanopore

Technologies, ONT) according to the manufacturer's instructions. Sequencing was performed on a GridION X5 device using a MinION R10.4.1 flow cell (FLO-MIN114; ONT).

Basecalling and Whole Genome Sequence Assembly

Basecalling of raw reads from each PCR aliquot was performed using the super-accurate basecalling model (v5.0) implemented in Dorado (v1.1.1) (https://github.com/nanoporetech/dorado) on the high-performance computing (HPC) cluster at the University of Bern (https://www.id.unibe.ch/hpc). Resulting FASTQ reads were filtered by length and retained if between 500 bp and 3000 bp using chopper (v0.9.2) (*4*). After concatenation of reads from sample replicates, merged reads were assembled into complete genomes using the FLU-Minion module of the Iterative Refinement Meta-Assembler (IRMA) software (*5*), applying default settings with elongation parameters enabled to improve recovery of 5′ and 3′ UTR regions. Post-assembly quality control, including gene-specific coverage, sequencing depth, and subtype confirmation, was performed using custom Python scripts. Subtyping results were further validated using BLAST and the BV-BRC web-based platform (*6*,*7*).

Sequence Alignment and Phylogenetic Analysis

An HA-H1 phylogenetic tree was constructed to classify the Swiss influenza A virus sequences into contemporary swine H1N1 lineages and to assess their genetic relationships with publicly available swine influenza A virus sequences from Switzerland and across Europe. All publicly available Swiss swH1N1 sequences (partial and full-length) and European sequences sampled between 2020 and 2024 were retrieved from the GISAID (https://gisaid.org/) and NCBI databases (accessed on 24 July 2025). Quality control was performed to remove redundant, short, and gapped sequences. Four top BLAST hit sequences showing >95% pairwise nucleotide similarity to the study sequences were additionally included. Codon-aligned multiple sequence alignments were generated using MAFFT v7 (*8*) and inspected and edited using AliView v1.30 (*9*). Pairwise nucleotide distances were estimated using the dist.dna function from the ape package and visualized using the pheatmap package in R v4.4.2 (*10*). Maximum-likelihood phylogenetic trees were inferred using IQ-TREE v3.0.1 with automated model selection and 100 bootstrap replicates (*11*). Trees were visualized and annotated using FigTree and Inkscape (https://inkscape.org/).

Variant Calling Analysis

To estimate between- and within-host low-frequency diversity, sequencing reads were aligned to segment-specific reference sequences for each sample using minimap2 v2.30 (*12*). Resulting BAM files were processed using samtools v1.3 for sorting, indexing, and format conversion (*13*). Single-nucleotide variants (SNVs) were identified using iVar v1.24.4 (*14*) and filtered to retain variants marked with a "PASS" flag, an alternative allele frequency ≥5%, and a p-value <0.05. Ambiguous calls (e.g., –n, +n) and indels were excluded. Codon positions and translated amino acids were determined using segment-specific GFF3 files generated with gfflu v0.0.2. (https://github.com/CFIA-NCFAD/gfflu.git). Processed SNVs were analyzed in Python 3 and visualized using the UpSetPlot v0.9.0 package (*15*).

Comparison of Antigenic Variation to Vaccine Strain and Key Mutations

To assess variation within defined HA antigenic regions (Sa, Sb, Ca1, Ca2, and Cb (*16*–*18*)), amino acid sequences of the study viruses were compared with those of WHO-recommended human influenza vaccine strains for the 2023/2024 season: A/Victoria/4897/2022/H1N1 (EPI_ISL_16714268) and A/Wiscosin/67/2022/H1N1 (EPI_ISL_15928563). An additional human seasonal H1N1 HA sequence from Switzerland sampled in 2023 was included for comparison. HA amino acid alignments were converted to the recommended HA-H1 numbering scheme using the HA Subtype Numbering Conversion Tool (*19*). Phenotypically or epidemiologically relevant mutations were further identified by querying sequences against the GISAID FluServer database, which compiles curated mutation information from the literature (https://flusurver.bii.a-star.edu.sg/).

Data Availability

Raw sequencing reads used in this work are available upon request. All swine-origin influenza A sequences generated in this study are publicly available in GISAID under accession numbers EPI_ISL_19132700, EPI_ISL_19132714, EPI_ISL_19132715, EPI_ISL_19132719, and EPI_ISL_19132751. The human-origin influenza A variant virus sequence generated in this study is available under EPI_ISL_19132752 (A/Switzerland/H0001/2023). Scripts used in this work can be accessed via the following link: https://doi.org/10.5281/zenodo.17019965.

References

1. Lechmann J, Szelecsenyi A, Bruhn S, Harisberger M, Wyler M, Bachofen C, et al. The Swiss national program for the -surveillance of influenza A viruses in pigs and humans: genetic variability and zoonotic transmissions from 2010– 2022. Schweiz Arch Tierheilkd. 2025;167:600–16. PubMed https://doi.org/10.17236/sat00466

2. Nagy A, Černíková L, Kunteová K, Dirbáková Z, Thomas SS, Slomka MJ, et al. A universal RT-qPCR assay for "One Health" detection of influenza A viruses. PLoS One. 2021;16:e0244669. PubMed https://doi.org/10.1371/journal.pone.0244669

3. Licheri M, Mwanga M, Licheri MF, Graaf-Rau A, Sägesser C, Bittel P, et al. Optimized high-throughput whole-genome sequencing workflow for surveillance of influenza A virus. Genome Med. 2025;17:103. PubMed https://doi.org/10.1186/s13073-025-01512-x

4. De Coster W, Rademakers R. NanoPack2: population-scale evaluation of long-read sequencing data. Bioinformatics. 2023;39:btad311. PubMed https://doi.org/10.1093/bioinformatics/btad311

5. Shepard SS, Meno S, Bahl J, Wilson MM, Barnes J, Neuhaus E. Viral deep sequencing needs an adaptive approach: IRMA, the iterative refinement meta-assembler. BMC Genomics. 2016;17:708. PubMed https://doi.org/10.1186/s12864-016-3030-6

6. Altschul SF, Gish W, Miller W, Myers EW, Lipman DJ. Basic local alignment search tool. J Mol Biol. 1990;215:403–10. PubMed https://doi.org/10.1016/S0022-2836(05)80360-2

7. Olson RD, Assaf R, Brettin T, Conrad N, Cucinell C, Davis JJ, et al. Introducing the Bacterial and Viral Bioinformatics Resource Center (BV-BRC): a resource combining PATRIC, IRD and ViPR. Nucleic Acids Res. 2023;51(D1):D678–89. PubMed https://doi.org/10.1093/nar/gkac1003

8. Yamada KD, Tomii K, Katoh K. Application of the MAFFT sequence alignment program to large data-reexamination of the usefulness of chained guide trees. Bioinformatics. 2016;32:3246–51. PubMed https://doi.org/10.1093/bioinformatics/btw412

9. Larsson A. AliView: a fast and lightweight alignment viewer and editor for large datasets. Bioinformatics. 2014;30:3276–8. PubMed https://doi.org/10.1093/bioinformatics/btu531

10. Paradis E, Schliep K. ape 5.0: an environment for modern phylogenetics and evolutionary analyses in R. Bioinformatics. 2019;35:526–8. PubMed https://doi.org/10.1093/bioinformatics/bty633

11. Minh BQ, Schmidt HA, Chernomor O, Schrempf D, Woodhams MD, von Haeseler A, et al. IQ-TREE 2: new models and efficient methods for phylogenetic inference in the genomic era. Mol Biol Evol. 2020;37:1530–4. PubMed https://doi.org/10.1093/molbev/msaa015

12. Li H. Minimap2: pairwise alignment for nucleotide sequences. Bioinformatics. 2018;34:3094–100. PubMed https://doi.org/10.1093/bioinformatics/bty191

13. Danecek P, Bonfield JK, Liddle J, Marshall J, Ohan V, Pollard MO, et al. Twelve years of SAMtools and BCFtools. Gigascience. 2021;10:giab008. PubMed https://doi.org/10.1093/gigascience/giab008

14. Grubaugh ND, Gangavarapu K, Quick J, Matteson NL, De Jesus JG, Main BJ, et al. An amplicon-based sequencing framework for accurately measuring intrahost virus diversity using PrimalSeq and iVar. Genome Biol. 2019;20:8. PubMed https://doi.org/10.1186/s13059-018-1618-7

15. Lex A, Gehlenborg N, Strobelt H, Vuillemot R, Pfister H. UpSet: visualization of intersecting sets. IEEE Trans Vis Comput Graph. 2014;20:1983–92. PubMed https://doi.org/10.1109/TVCG.2014.2346248

16. Caton AJ, Brownlee GG, Yewdell JW, Gerhard W. The antigenic structure of the influenza virus A/PR/8/34 hemagglutinin (H1 subtype). Cell. 1982;31:417–27. PubMed https://doi.org/10.1016/0092-8674(82)90135-0

17. Van Reeth K, Parys A, Gracia JCM, Trus I, Chiers K, Meade P, et al. Sequential vaccinations with divergent H1N1 influenza virus strains induce multi-H1 clade neutralizing antibodies in swine. Nat Commun. 2023;14:7745. PubMed https://doi.org/10.1038/s41467-023-43339-3

18. Gerhard W, Yewdell J, Frankel ME, Webster R. Antigenic structure of influenza virus haemagglutinin defined by hybridoma antibodies. Nature. 1981;290:713–7. PubMed https://doi.org/10.1038/290713a0

19. Burke DF, Smith DJ. A recommended numbering scheme for influenza A HA subtypes. PLoS One. 2014;9:e112302. PubMed https://doi.org/10.1371/journal.pone.0112302

Appendix Table 1. Reaction conditions for the detection of influenza A virus by real-time RT-qPCR

Step	Temperature (°C)	Time	Cycles
Reverse transcription	50	10 min	1
Heat-inactivation	95	30 s	1
Denaturing	95	5 s	45
Annealing/Extension	60	30 s	45
Cooling	40	30 s	1

Appendix Table 2. Phenotypically relevant mutations identified in Swiss swH1N1 sequences using the GISAID FluServer tool (http://flusurver.bii.a-star.edu.sg).

Gene	Mutation	Number of sequences with mutation	Phenotypic significance	Reference
HA	S173N	6	Host specificity shift	doi.org/10.1128/jvi.00221-10
M2	L26I	6	Strong drug resistance (neuraminidase inhibitors and adamantanes)	doi.org/10.1016/j.antiviral.2006.10.004
	V27A	6	Strong drug resistance (amantadine)	doi.org/10.1016/j.virol.2005.07.003
	S31N	6	Strong drug resistance (amantadine)	doi.org/10.3201/eid1506.081357
PB2	D701N	6	Virulence and host specificity	doi.org/10.1128/jvi.00666-07

Appendix Table 3. Summary of single-nucleotide variants detected across viral gene segments

Sample	Ref pos	Gene	Ref codon	Alt. codon	Alt. freq	AA position	Ref AA	Alt AA
A0001	1184	NP	aac	aGc	0.12	395	N	S
A0001	1771	PA	tag	Cag	0.22	591	*	Q
A0001	1106	PB2	agg	aAg	0.12	369	R	K
A0001	1447	PB2	aca	Gca	0.13	483	T	A
A0002	788	NS	act	aTt	0.07	263	T	I
A0002	571	PB1	gta	Ata	0.50	191	V	I
A0002	1447	PB2	aca	Gca	0.22	483	T	A
A0003	515	HA	gga	gAa	0.46	172	G	E
A0003	544	NS	gtc	Atc	0.10	182	V	I
A0003	1447	PB2	aca	Gca	0.16	483	T	A
A0004	139	NS	ggc	Agc	0.06	47	G	S
A0004	313	NS	ctc	Atc	0.10	105	L	I
A0004	1447	PB2	aca	Gca	0.13	483	T	A
A0004	1940	PB2	ata	aCa	0.36	647	I	T
A0005	515	HA	gga	gAa	0.44	172	G	E
A0005	940	HA	ccc	Tcc	0.14	314	P	S
A0005	1447	PB2	aca	Gca	0.10	483	T	A
A0005	1982	PB2	aca	aAa	0.10	661	T	K
H0001	515	HA	gga	gAa	0.13	172	G	E
H0001	389	NP	act	aAt	0.06	130	T	N
H0001	928	NP	agc	Ggc	0.06	310	S	G
H0001	1738	PA	gaa	Aaa	0.16	580	E	K
H0001	1771	PA	cag	Tag	0.19	591	Q	*
H0001	1928	PA	aaa	aGa	0.06	643	K	R
H0001	1447	PB2	aca	Gca	0.12	483	T	A
H0001	1940	PB2	ata	aCa	0.11	647	I	T

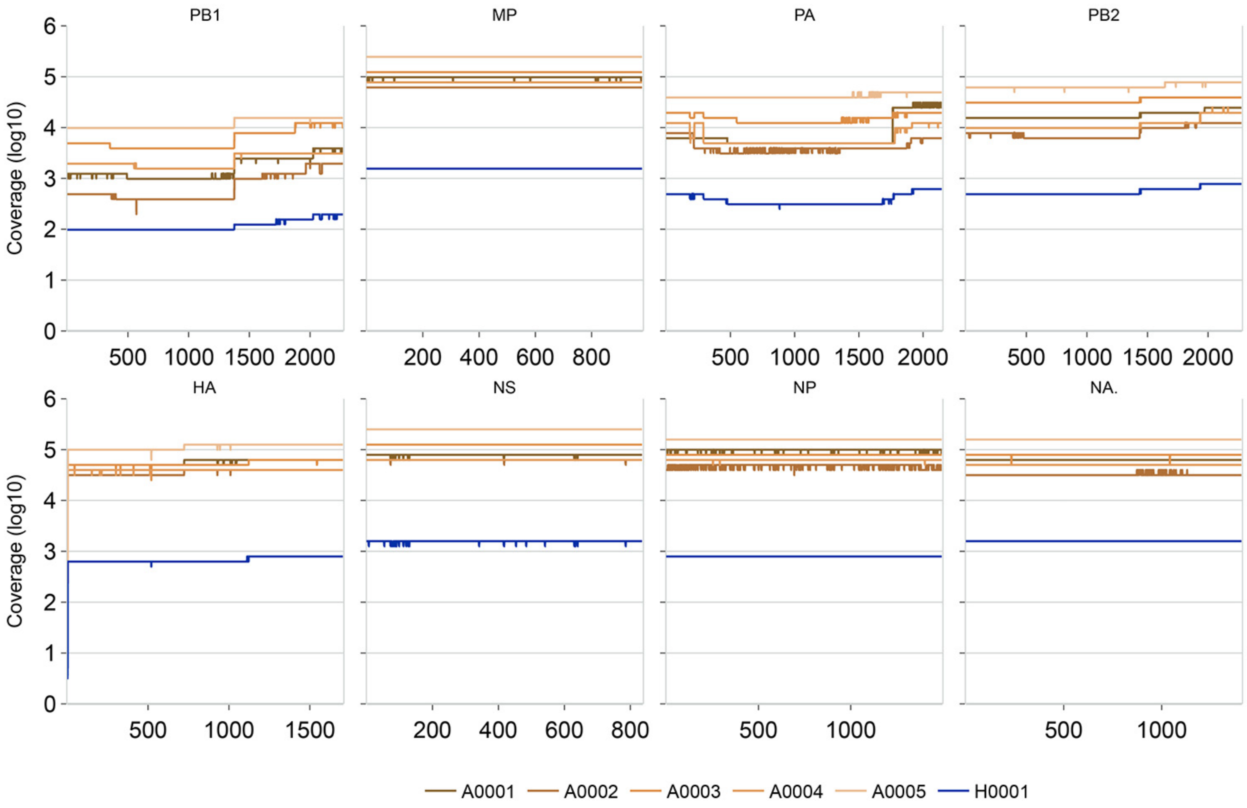

Appendix Figure 1. Sample-specific coverage showing read depth per influenza A virus gene segment. Pig samples (A0001–A0005) are shown in varying shades of orange/brown and the human sample (H0001) is shown in blue.

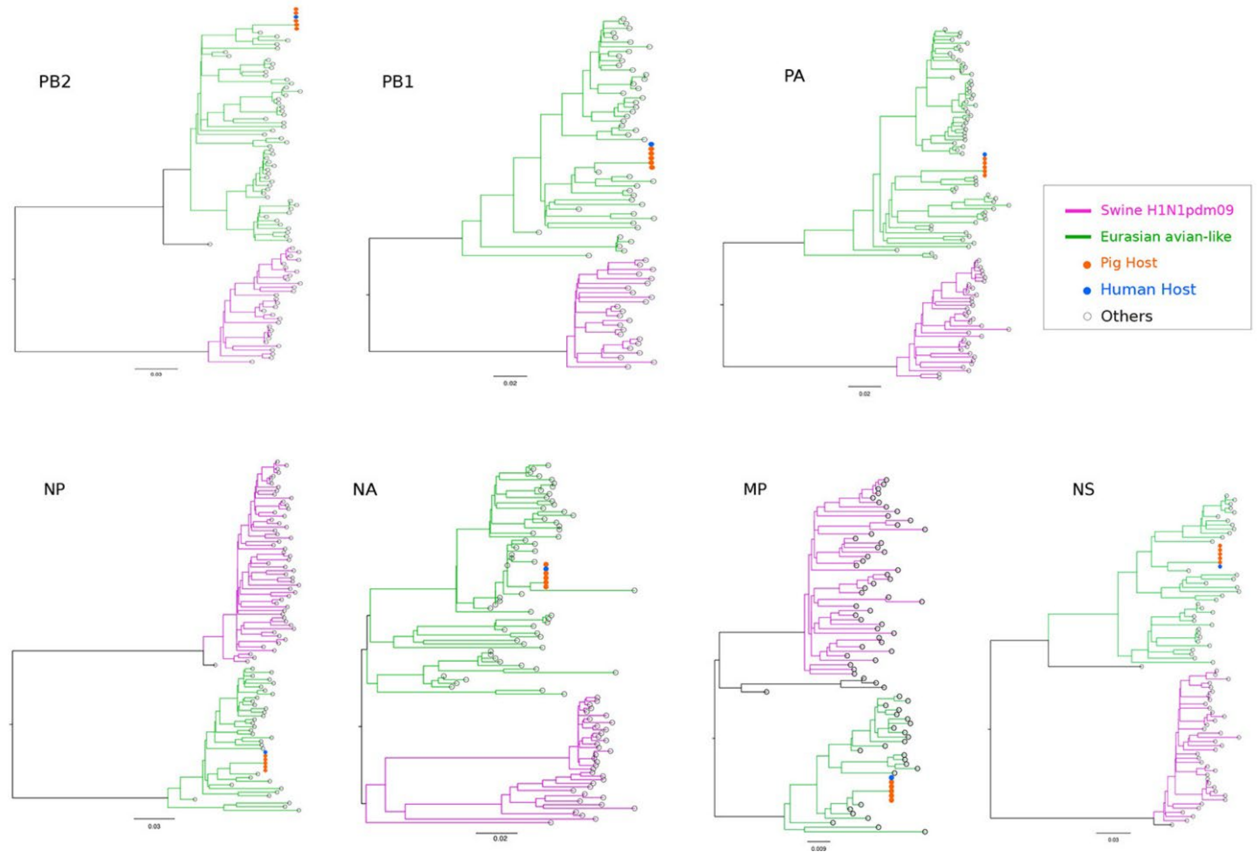

Appendix Figure 2. Segment-specific maximum-likelihood phylogenetic trees showing lineage placement of the PB2, PB1, PA, NP, NA, MP, and NS gene segments of the study viruses. Sequences from pig and farmer samples are represented by orange- and blue-shaded tip nodes, respectively. Unshaded tips represent Eurasian avian-like (green) and H1N1pdm09 (purple) lineages from swH1N1 viruses isolated in Europe. All study sequences clustered within the Eurasian avian-like lineage.

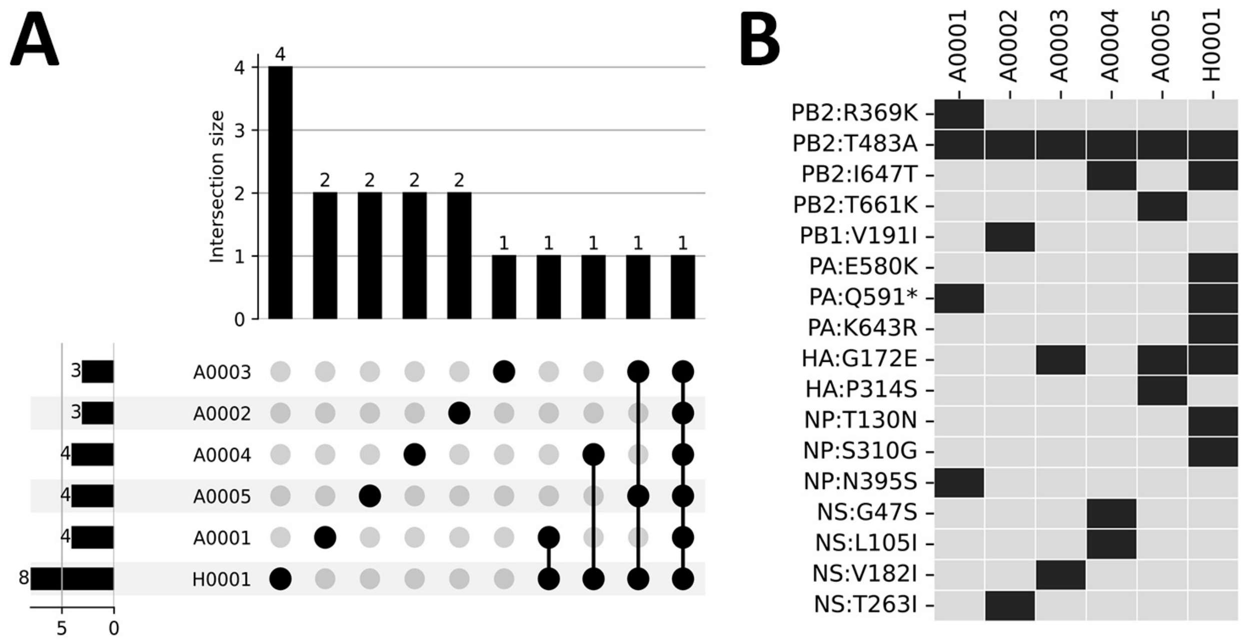

Appendix Figure 3. Single-nucleotide variants (SNVs) detected in human and pig samples. (A) Distribution of unique and overlapping SNVs between samples. The single bar on the left represents the total number of SNVs detected per sample. Intersection bars indicate the number of shared SNVs between samples, as denoted by dots in the matrix. B) Heatmap of non-synonymous amino acid substitutions arising from detected SNVs (black). The y-axis indicates segment-specific amino acid positions, and the x-axis denotes the samples in which each substitution was detected. Pig samples are labeled A0001–A0005, and the human sample is labeled H0001. Asterisk represents SNV that results in a stop codon.
